# Pulmonary Infection Is an Independent Risk Factor for Long-Term Mortality and Quality of Life for Sepsis Patients

**DOI:** 10.1155/2016/4213712

**Published:** 2016-12-05

**Authors:** Xiao-Li He, Xue-Lian Liao, Zhi-Chao Xie, Li Han, Xiao-Lei Yang, Yan Kang

**Affiliations:** Department of Critical Care Medicine, West China Hospital, Sichuan University, Chengdu, China

## Abstract

*Background.* Long-term outcomes (mortality and health-related quality of life) of sepsis have risen as important indicators for health care. Pulmonary infection and abdominal infection are the leading causes of sepsis. However, few researches about long-term outcomes focused on the origin of sepsis. Here we aim to study the clinical differences between pulmonary-sepsis and abdominal-sepsis and to investigate whether different infection foci were associated with long-term outcomes.* Methods.* Patients who survived after hospital discharge were followed up by telephone interview. Quality of life (QoL) was assessed using the EuroQol 5-dimension (EQ5D) questionnaire.* Results.* Four hundred and eighty-three sepsis patients were included, 272 (56.3%) had pulmonary-sepsis, and 180 (37.3%) had abdominal-sepsis. The overall ICU and one-year mortality rates of the cohort were 17.8% and 36.1%, respectively. Compared with abdominal-sepsis, pulmonary-sepsis patients had older age, higher APACHE II, higher ICU mortality (31.7% versus 12.6%), and one-year mortality (45.4% versus 24.4%), together with worse QoL. Age, septic shock, acute renal failure, fungus infection, anion gap, and pulmonary infection were predictors for one-year mortality and pulmonary infection was a risk factor for poor QoL.* Conclusions.* Pulmonary-sepsis showed worse outcome than abdominal-sepsis. Pulmonary infection is a risk factor for one-year mortality and QoL after sepsis.

## 1. Introduction

Despite advances in organ support and guidelines for sepsis management, the incidence of sepsis is still increasing [1–5]. Sepsis is the leading cause of death among hospitalized patients [6], mortality of which ranging from 20 to 80%. Sepsis survivors also suffered persistent reduction in long-term health-related quality of life (HR-QoL), such as depression, morbidity, and cognitive impairment [7–9]. This reduction can persist up to 5 years after hospital discharge [10]. For better evaluation of the long-term outcomes of sepsis, we should focus not only on its long-term mortality but also on HR-QoL.

More and more researches have showed that the EQ5D questionnaire can be used in critically ill patients to evaluate long-term HR-QoL [11–13]. The EQ5D questionnaire includes five dimensions, namely, mobility ability, self-care, usual activity, pain/discomfort, and anxiety/depression. Each dimension has three different levels, separately none, moderate, and severe problem. An EQ5D index can be obtained based on the EQ5D questionnaire via a Japanese version conversion table [14]. The visual analog scale (VAS), as a part of the questionnaire, is also used. The EQ-VAS, a score ranging from 0 to 100, can subjectively reflect the health state of patients, where 0 means the worst state and 100 the best [14].

Pneumonia is one of the most common reasons for admission to intensive care units (ICUs). Studies have revealed that pneumonia is the primary kind of sepsis [15–17]. Kim and his colleagues' study showed that pneumonia is associated with higher mortality when compared to other infection sources [18].

Abdominal infection is another common indication for admission to ICU, and abdomen is the second popular site of invasive infection among critically ill patients [19–21]. Poor control of abdominal infection frequently results in abdominal-sepsis [22].

Lung and abdomen are the most common sources of sepsis [4, 12]. Existing research on the outcome of sepsis according to the infection foci is sparse and information about difference between pulmonary-sepsis and abdominal-sepsis is still limited. Our study focuses on elucidating the clinical difference between pulmonary-sepsis and abdominal-sepsis, the variation in long-term mortality, and QoL of different sepsis origin and identifying the predictors of long-term mortality and QoL for sepsis survivors.

## 2. Materials and Methods

### 2.1. Study Population

This prospective cohort study was carried out among patients admitted to the combined surgical, respiratory, and medical intensive care units of West China hospital of Sichuan University (from December 2013 to December 2014). Patients diagnosed with sepsis as the primary cause for ICU administration were identified and enrolled within the first 24 hours. Patients younger than 18 years were excluded, and so were patients with a length of ICU stay less than 24 hours. If the patient was admitted to the ICU more than once, only the first sepsis episode was enrolled. HR-QoL was assessed using the EQ5D questionnaire. Permission to perform the follow-up study was granted by the Clinical Trials and Biomedical Ethics Committee of West China Hospital.

### 2.2. Definitions, Data Collection, and Outcome Measures

Sepsis was defined as at least two systemic inflammatory syndrome criteria together with infection evidence [23]. At least one of the following criteria was required for diagnosis of pneumonia: (1) clinical features including fever (>38°C) or hypothermia (≤35°C), new cough wherever with or without sputum, dyspnea, pleuritic chest pain, or changed respiratory sounds; (2) radiographic evidence of lung infection with a newly onset or changed infiltrate focus based on the guidelines of German College of Pulmonology [24]. Abdominal infection includes bacterial liver abscess, acute peritonitis, acute binary tract infection cholecystitis, and acute pancreatitis complicated with secondary bacterial infections.

Demographic characteristics, infection site, type of infection (G+/G−, fungus, or virus), laboratory results in the first 24 hours, comorbidities, length of ICU and hospital stay, ICU administration strategy such as mechanical ventilation, continuous renal replacement therapy (CRRT), and use of vasoactive agent were recorded. The Acute Physiology and Chronic Health Evaluation (APACHE) II score [25] and Sepsis-related Organ Failure Assessment (SOFA) score [26] in the first 24 hours of ICU admission, were also collected to assess the severity of illness. Primary outcome was one-year mortality, and secondary outcome was one-year QoL assessed* via* EQ5D. All clinical data were obtained from the Hospital Information System of West China Hospital and follow-up information was recorded by the telephone interviewer.

### 2.3. Statistical Analysis

Statistical analysis was conducted in SPSS software version 19.0 (SPSS Inc., Chicago, IL, USA). The Kolmogorov-Smirnov test was used to assess the data normality. Quantitative data exhibiting normal distributions were expressed as mean and standard deviation (SD) or, otherwise, presented as median with 25th and 75th percentile on rejection of the normality hypothesis. Students'* t*-test was used for the analysis of normally distributed continuous variables. The Mann–Whitney test was used to explore the difference between the independent groups when the data was not normally distributed. For categorical variables, the *χ*2 (for large sample) or Fisher's exact test (for small sample) was applied appropriately to calculate the difference between groups. Backward stepwise binary logistic regression was conducted to find predictors for one-year mortality and QoL after the sepsis episode. All the tests were two-tailed and a *p* value less than 0.05 was considered statistically significant. Missing data were handled* via* simple deletion method and patients lost to follow-up were excluded when analyzing one-year mortality and quality of life.

## 3. Results

**Figure 1:**
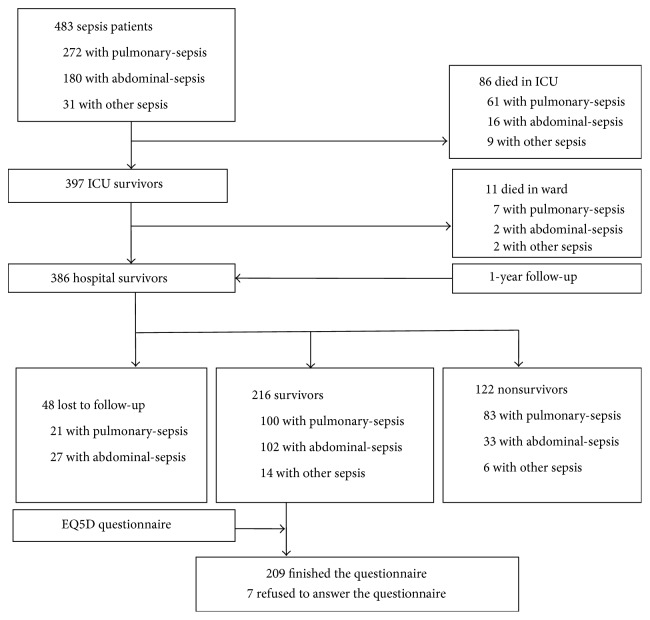
Flow chart of the follow-up study. EQ5D, EuroQol-5D.

Study flow was presented in Figure 1. Of the 483 patients, 86 died in ICU and 11 died in ward. Three hundred and eighty-six hospital survivors were followed up by telephone one year after ICU discharge. Forty-eight patients were lost to follow-up. Of the others, 216 patients survived one year after ICU discharge, and then EQ5D questionnaire was used for the assessment of QoL for 1-year survivors and 209 of them finished the questionnaire (Figure 1).

### 3.1. Demographic Characteristics of the Sepsis Cohort

Among the 483 sepsis patients, pulmonary-sepsis (56.3%, *n* = 272) was the most common type of sepsis, followed by abdominal infection (37.3%, *n* = 180). The mean age of the sepsis cohort was 60.3 years, and the average APACHE II score was 21.5. For the whole study population, the incidence of sepsis, severe sepsis, and septic shock was 7.7%, 27.5%, and 64.8%, respectively. Pulmonary-sepsis patients were much older (63.7 years old) than abdominal-sepsis patients (56.7 years old) (*p* = 0.000) and had higher APACHE II score (23.0 versus 18.6, *p* = 0.000). The SOFA score of pulmonary-sepsis (median 9, IQR 7–12) was significantly worse than that of abdominal-sepsis (median 7, IQR 5–11). Pulmonary-sepsis had a higher Charlson Comorbidity Index. Fungal or viral infection was more likely to be identified in the pneumonia-induced sepsis population. Pulmonary-sepsis was more prone to develop acute renal failure (17.6%, *p* = 0.043) and had greater need for CRRT during the whole ICU stay period (20.2% and 11.1%, *p* = 0.014). Pulmonary-sepsis had longer MV days and length of ICU stay (*p* = 0.000). Demographic characteristics of the study cohort were presented in Table 1.

### 3.2. Mortality of the Sepsis Cohort

**Figure 2:**
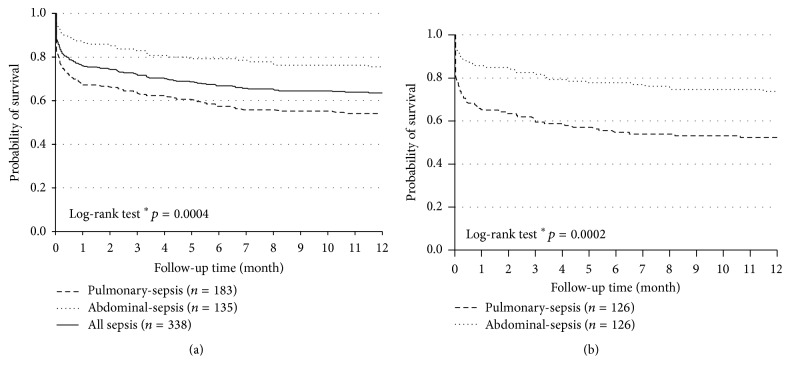
Probability of one-year survival for ICU survivors. (a) Probability of one-year survival for all ICU survivors of the unmatched cohorts. (b) Probability of one-year survival for ICU survivors of the age-matched cohorts. ^*∗*^ *p* value indicated for comparison between pulmonary-sepsis and abdominal-sepsis.

ICU mortality for all sepsis, pulmonary-sepsis, and abdominal-sepsis was 17.8% (*n* = 86), 22.4% (*n* = 61), and 8.9% (*n* = 16), respectively, and hospital mortality was 20.1% (*n* = 97), 25.0% (*n* = 68), and 10.0% (*n* = 18), respectively (Table 1). The overall 28-day mortality of the ICU survivors for all sepsis, pulmonary-sepsis, and abdominal-sepsis was 23.4% (*n* = 79), 31.7% (*n* = 58), and 12.6% (*n* = 17), respectively, and one-year mortality of ICU survivors was 36.1% (*n* = 122), 45.4% (*n* = 83), and 24.4% (*n* = 33), respectively (Table 1) (when analyzing one-year mortality, all sepsis *n* = 437, patients lost to follow-up were excluded). Kaplan-Meier curve also showed that patients with pulmonary-sepsis had higher one-year mortality than that of the patients with abdominal-sepsis (Figure 2(a)). Considering the older age and greater comorbidity burden on the pulmonary-sepsis cohort, we did an age-matched cohort study of ICU survivors to adjust the impact on long-term mortality. Similar results were obtained; that is, pulmonary-sepsis showed poor survival (Figure 2(b)). Background characteristics of the age-matched cohort were shown in Supplementary Table 1 in Supplementary Material available online at http://dx.doi.org/10.1155/2016/4213712.

### 3.3. One-Year QoL (EQ5D) of Sepsis Survivors

**Figure 3:**
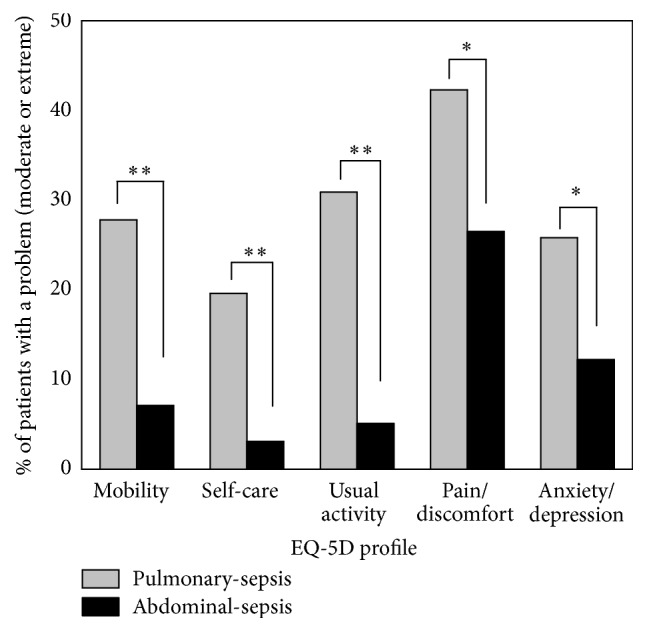
EQ5D profile in one-year survivors of pulmonary-sepsis and abdominal-sepsis. The EuroQol 5D profile is dichotomized into “no problems” and “moderate or extreme problems” 1 year after ICU discharge ^*∗*^ *p* < 0.05; ^*∗∗*^ *p* < 0.01.

The distribution of the five dimensions in the EQ5D questionnaire was described in Table 2. Of all the survivors who completed the EQ5D questionnaire, 18.7% had moderate to severe problem in mobility, 12.5% in self-care, 19.2% in pain/discomfort, 33.5% in anxiety/depression, and 19.1% in anxiety/depression. This showed that most patients had problems in the pain/discomfort dimension. The median EQ5D index was 0.848, and the median EQ-VAS was 80. Pulmonary-sepsis patients showed more problems than abdominal-sepsis patients in all the five dimensions (Figure 3, Table 2). Significant difference was found in both the EQ5D index and EQ-VAS (*p* = 0.001 for both). Pulmonary-sepsis patients showed worse one-year QoL (Table 2).

### 3.4. Risk Factors for One-Year Mortality

To find risk factors for one-year mortality, a total of 435 sepsis patients were involved in the analysis. Of them, 216 (49.7%) survived one year after ICU discharge. Nonsurvivors tended to be much older and had apparently higher APACHE II, SOFA, and Charlson Comorbidity Index (all *p* = 0.000). Greater ratio of patients in nonsurvivors was identified with fungal infection (*p* = 0.000). The incidence of septic shock was obviously higher in the nonsurvivor group (74.8%) than that of the survivor group (56.0%) (*p* = 0.000). One hundred and fifty-two of the 219 (69.7%) nonsurvivors had pulmonary-sepsis, which was much higher than the survivor group (46.3%). However, abdominal-sepsis was more frequently found in the survivor group (47.2% versus 22.9%, *p* = 0.000). Within the first 24 hours after admission to the ICU, there was a greater need of vasopressor use for the nonsurvivors (47.2% versus 29.6%, *p* = 0.000). Similarly, nonsurvivors were more prone to develop acute renal failure than survivors and had more requirements for CRRT. Mechanical days and length of hospital stay (*p* = 0.000) were also longer in the nonsurvivors group, but there was no difference in ICU LOS (*p* = 0.605). Laboratory parameters such as creatinine, plates, cystatin c, LDH, and anion gag were also worse in the nonsurvivors (Table 1).

Univariate analysis of the mortality showed age, APACHE II, SOFA, Charlson Comorbidity Index, malignancy, acute renal failure, pulmonary infection, fungus infection, septic shock, cystatin c, and anion gap as potential predictors for one-year mortality. After multivariate adjustment, age (OR = 1.025; 95% CI, 1.011–1.039), septic shock (OR = 2.533; 95% CI, 1.591–4.032), fungus infection (OR = 1.846; 95% CI, 1.160–2.938), acute renal failure (OR = 2.914; 95% CI, 1.525–5.568), anion gap (OR = 1.070; 95% CI, 1.025–1.117), and pulmonary infection (OR = 2.547; 95% CI, 1.513–4.288) were risk factors for one-year mortality (Table 3).

### 3.5. Predictors for One-Year Quality of Life

In order to find predictors for one-year QoL, QoL was evaluated by EQ5D index. EQ5D index less than 0.848 (median) was defined as poor QoL. Survivors were divided into poor and good QoL groups. Background characteristics were summarized in Table 4. Patients with poor QoL had higher APACHE II and Charlson Comorbidity Index, prolonged mechanical ventilation, longer ICU, and hospital LOS. Patients of the poor QoL group were more prone to suffer pulmonary infection (76.7% versus 47.2%), and 57.3% of them had pulmonary-sepsis, while only 25.8% of patients in the good QoL group had pulmonary-sepsis. Univariate analysis suggested that APACHE II, chronic heart failure, pulmonary infection, and tube extubation during the first 24 hours after admission to ICU were possible predictive factors of one-year QoL (Table 3). Multivariate logistic regression showed that pulmonary infection (OR = 2.846, 95% CI (1.530–5.294)) was a risk factor of one-year QoL, while tube extubation during the first 24 hours (OR = 0.330, 95% CI (0.110–0.989)) was a protective factor (Table 3).

## 4. Discussion

This study showed that short- and long-term outcomes between patients with pulmonary-sepsis and abdominal-sepsis vary greatly. Our findings suggest that patients with pulmonary-sepsis were more prone to fungal infection, acute renal failure requiring CRRT, prolonged mechanical ventilation, longer ICU and hospital stays, and higher in-hospital and one-year mortality than the abdominal-sepsis group. In addition, the pulmonary-sepsis cohort had worse QoL indicators after hospital discharge. To our knowledge, our follow-up study was one of the few researches to investigate the clinical difference of the most frequently identified sepsis source, including short-term and long-term mortality, together with QoL.

Our study found that age, septic shock, acute renal failure, fungal infection, anion gap, and pulmonary infection were potential risk factors for increased one-year mortality. It is not surprising that older age positively correlates with higher long-term mortality. Septic shock is the most severe stage of sepsis and long-term outcome of septic shock was poor. Nesseler et al. [27] reported that 6-month mortality of septic shock was 45%. Harris et al. [28] found that critically ill patients with acute kidney injury had higher one-year mortality, and it is reasonable to speculate that there was higher one-year mortality in patients with acute renal failure. Fungal infection usually occurs in patients with immunosuppression and was associated with increased hospital mortality [29]. Previous researches have shown that anion gap increases in 72% of critically ill patients, and elevated AG has been found to be associated with mortality in serious diseases, including critical illness [30–35].

The research revealed that pulmonary infection was associated with increased short-term and long-term mortality which was in accordance with previous studies. Mansur et al.'s study [36] reported a higher 90-day mortality in pulmonary-sepsis than abdominal-sepsis. Kim et al. [18] reported significantly higher 28 d mortality of pneumonia (41%) than non-pulmonary-sepsis (30%), and pneumonia was demonstrated to be a risk factor for 28-day mortality. In our study cohort, we found that pulmonary-sepsis patients were much older and had higher APACHE II, SOFA score, and Charlson Comorbidity Index. Comorbidities and laboratory parameters on admission of the sepsis cohort were shown in Table 1. Consistent with our study, the PAO_2_/FiO_2_ and PaO_2_ of pulmonary-sepsis patients were worse than other sepsis source patients and previous research had already validated Pao_2_/FiO_2_ as a biomarker for prognosis of sepsis such as mortality [18]. What is more, patients in the pulmonary-sepsis cohort were significantly older and had a higher rate of renal failure, thus explaining their higher APCHE II scores. The SOFA score of pulmonary-sepsis was apparently higher than that of the abdominal group (*p* = 0.003); however, this difference disappeared when comparing the nonpulmonary SOFA scores (*p* = 0.125); that is, the difference of SOFA scores between groups was primarily caused by the pulmonary component which can be explained by pneumonia. Pneumonia patients had a greater probability to have chronic pulmonary disease (32% and 11.7%, *p* = 0.000). COPD was the most common chronic pulmonary disease and the quality of life for patients with COPD was apparently impaired [37]. Greater portion of patients with cardiovascular disease, cerebrovascular disease, chronic pulmonary disease, and chronic kidney disease in the pulmonary-sepsis group also contributed to high long-term mortality [38–41]. In order to eliminate the impact of older age and age-associated diseases on the pulmonary-sepsis cohort, an age-matched cohort analysis was conducted. Survival analysis of both the unmatched and the matched cohorts showed greater mortality in the pulmonary-sepsis group (Figures 2(a) and 2(b)).

Quality of life for sepsis was impaired [27, 42]. Patients with poor QoL were much older, had higher APACHE II, SOFA, and Charlson Comorbidity Index, and had prolonged mechanical ventilation days and ICU and hospital LOS (Table 4). Chronic heart failure was also found more commonly in the poor QoL group. A total of 57.3% of the 103 survivors in the poor QoL group were diagnosed with pulmonary-sepsis when admitted to the ICU and 76.7% of survivors with poor QoL suffered pulmonary infection in ICU (Table 4). In accordance with data shown in Table 1, pulmonary-sepsis cases had older age, higher APACHE II and SOFA score, and greater comorbidity burden (Table 1, Figure 2). Patients with tube weaning in the first 24 hours had better QoL, since these patients tended to be less serious, had less need for mechanical ventilation, and could soon recover from the sepsis attack. Pulmonary infection was already confirmed to be a risk factor for 28 d mortality [18]. Our study was the first to confirm its role in decreased QoL.

There were several limitations in our study. Firstly, this follow-up study was a single-center study conducted in a teaching hospital. This study design would result in lack of representativeness. Patients admitted to our hospital appeared to be much more serious, and they were much older and had more complications than patients admitted to ICUs of other hospitals, resulting in an overestimation of mortality. Moreover, a majority of patients were transferred from other hospitals and patients fulfilling the sepsis criteria at the onset of disease might fail to be diagnosed as having sepsis. These could all lead to selection bias. Secondly, the evaluation of GCS was inaccurate due to the use of sedation and approximately half of the cohort did not have a measurement of lactate during the 24 hours. Thirdly, Tibetan patients who could not speak Mandarin were excluded for language barrier, increasing the rate of patients lost to follow-up. Multicenter studies with larger samples were needed to confirm the study results.

## 5. Conclusions

Patients diagnosed with sepsis show ongoing mortality after the sepsis episode, with only 63.9% surviving one year after ICU discharge. Pulmonary-sepsis had worse short-term and long-term outcomes, including ICU/hospital mortality, one-year mortality, and one-year quality of life. Pulmonary infection is a risk factor for one-year mortality and is associated with decreased health-related quality of life.

## Supplementary Material

Of the age-matched sepsis cohort, there was no difference in age, Charlson Comorbidity Index and SOFA score. However, pulmonary sepsis had apparently higher APACHE II(22.1 vs 18.6), prolonged mechanical ventilation days, longer ICU and hospital days. Abdominal-sepsis patients were more prone to develop septic shock.

## Figures and Tables

**Table 1:** Demographic characteristics of the sepsis cohort.

Variables	All sepsis	Pulmonary-sepsis	Abdominal-sepsis	*p* ^a^	Nonsurvivors	Survivors	*p* ^b^
	*n* = 483	*n* = 272	*n* = 180		*n* = 219	*n* = 216	
Age, mean (SD)	60.3 (16.2)	63.7 (15.3)	56.7 (16.1)	0.000^*∗∗*^	64.2 (15.3)	56.7 (16.6)	0.000^*∗∗*^
Male sex, *n*%	319 (66.0)	186 (67.6)	114 (63.3)	0.363	140 (64.2)	147 (68.1)	0.418
APACHE II, mean (SD)	21.5 (7.9)	23.0 (6.9)	18.6 (8.0)	0.000^*∗∗*^	24.4 (7.5)	19.3 (7.4)	0.000^*∗∗*^
SOFA	8 (6–11)	9 (7–12)	7 (5–11)	0.003^*∗∗*^	10 (7–12)	7 (5–10)	0.000^*∗∗*^
SOFA, nonpulmonary	6 (4–8)	6 (4–8)	5 (3–8)	0.125	7 (5–9)	5 (3–8)	0.000^*∗∗*^
Charlson Comorbidity Index	3 (1–5)	4 (2–5)	2 (1–4)	0.000^*∗∗*^	4 (2–5)	2 (1–4)	0.000^*∗∗*^
Comorbidities							
Cardiovascular disease	182 (37.7)	121 (44.5)	51 (28.3)	0.001^*∗∗*^	98 (45.0)	76 (35.2)	0.040^*∗*^
Cerebrovascular disease	47 (9.7)	35 (12.9)	9 (5.0)	0.006^*∗∗*^	25 (11.5)	19 (8.8)	0.427
Diabetes mellitus	88 (18.2)	55 (20.2)	25 (13.9)	0.102	47 (21.6)	36 (16.7)	0.223
Peripheral vascular disease	65 (13.5)	46 (16.9)	18 (10.0)	0.040^*∗*^	29 (13.3)	30 (13.9)	0.889
Digestive and liver disease	140 (29.0)	70 (25.7)	66 (36.7)	0.016^*∗*^	68 (31.2)	61 (28.2)	0.529
Malignancy	71 (14.7)	41 (15.1)	26 (14.4)	0.893	41 (18.8)	25 (11.6)	0.045^*∗*^
Chronic pulmonary disease	110 (22.8)	87 (32.0)	21 (11.7)	0.000^*∗∗*^	64 (29.4)	36 (16.7)	0.002^*∗∗*^
Chronic kidney disease	40 (8.3)	31 (11.4)	7 (3.9)	0.005^*∗∗*^	22 (10.1)	16 (7.4)	0.396
Other diseases	110 (22.8)	76 (27.9)	30 (16.7)	0.006^*∗∗*^	58 (26.6)	40 (20.4)	0.141
Pathogen, *n*%							
Gram positive	40 (8.3)	19 (7.0)	16 (8.9)	0.591	12 (5.6)	16 (7.6)	0.439
Gram negative	265 (54.9)	152 (55.9)	97 (53.9)	0.495	121 (56.3)	111 (52.6)	0.496
Fungus	175 (36.2)	122 (44.9)	42 (23.3)	0.000^*∗∗*^	98 (45.0)	60 (27.8)	0.000^*∗∗*^
Virus	20 (4.1)	16 (5.9)	3 (1.7)	0.031^*∗*^	7 (3.2)	9 (4.2)	0.621
Type of sepsis, *n*%				0.084			0.000^*∗∗*^
Sepsis	37 (7.7)	23 (8.5)	11 (6.1)	0.467	5 (2.3)	23 (10.6)	0.000^*∗∗*^
Severe sepsis	133 (27.5)	83 (30.5)	44 (24.4)	0.166	50 (22.9)	72 (33.3)	0.019^*∗*^
Septic shock	313 (64.8)	166 (61.0)	125 (69.4)	0.072	163 (74.8)	121 (56.0)	0.000^*∗∗*^
Origin of sepsis, *n*%							
Pulmonary	272 (56.3)	—	—	—	152 (69.7)	100 (46.3)	0.000^*∗∗*^
Abdominal	180 (37.3)	—	—	—	50 (22.9)	102 (47.2)	0.000^*∗∗*^
Other	31 (6.4)	—	—	—	16 (7.3)	15 (6.9)	1.000
Pulmonary infection^c^, *n*%	346 (71.6)	272 (100)	62 (34.4)	0.000^*∗∗*^	172 (78.9)	135 (62.5)	0.000^*∗∗*^
ICU treatment within 24 h hours, *n*%							
IPPV	420 (87.0)	228 (83.8)	164 (91.1)	0.033^*∗*^	195 (90.3)	180 (88.7)	0.634
NPPV	34 (7.0)	24 (8.8)	8 (4.4)	0.092	15 (6.9)	16 (7.9)	0.852
Tracheal extubation^d^	40 (8.3)	8 (2.9)	30 (16.9)	0.000^*∗∗*^	12 (5.5)	26 (12.0)	0.017^*∗*^
Vasopressor	181 (37.5)	91 (33.5)	74 (41.7)	0.090	103 (47.2)	64 (29.6)	0.000^*∗∗*^
ARF, *n*%	74 (15.3)	48 (17.6)	19 (10.6)	0.043^*∗*^	54 (24.8)	17 (7.9)	0.000^*∗∗*^
CRRT, *n*%	83 (17.2)	55 (20.2)	20 (11.1)	0.014^*∗*^	60 (27.5)	21 (9.7)	0.000^*∗∗*^
MV, d	8 (3–16)	11 (5–20.8)	5 (2–11)	0.000^*∗∗*^	11 (4–21)	6 (3–13)	0.000^*∗∗*^
ICU LOS, d	13 (6–23)	14 (7–3)	8.5 (4–19)	0.000^*∗∗*^	14 (5–24)	12 (6–22)	0.605
Hospital LOS, d	23 (13–39)	24 (13–37)	23 (13–44)	0.649	18 (8.8–33)	28 (17–47)	0.000^*∗∗*^
ICU mortality	86 (17.8)	61 (22.4)	16 (8.9)	0.000^*∗∗*^	—	—	—
Hospital mortality	97 (20.1)	68 (25.0)	18 (10.0)	0.000^*∗∗*^	—	—	—
28-day mortality^e^	79 (23.4)	58 (31.7)	17 (12.6)	0.000^*∗∗*^	—	—	—
1-year mortality^f^	122 (36.1)	83 (45.4)	33 (24.4)	0.000^*∗∗*^	—	—	—
Laboratory parameters on admission							
PLT 10^9^	140 (88–219)	137 (85–200)	158 (89–248)	0.041^*∗*^	133 (72–203)	152 (92–232)	0.027^*∗*^
Albumin g/L, mean (SD)	26.9 (6.8)	29.1 (6.1)	23.4 (6.7)	0.000^*∗∗*^	27.5 (23.3–31.6)	26.6 (21.7–31.1)	0.071
Creatinine, *μ*mol/L	82 (57–142)	84 (59–171)	79 (54–125)	0.072	98 (61–197)	73 (54–119)	0.001^*∗∗*^
Cystatin c, mg/L	1.1 (0.9–1.9)	1.3 (0.9–2.2)	0.9 (0.7–1.4)	0.000^*∗∗*^	1.4 (1.0–2.3)	1.0 (0.8–1.4)	0.000^*∗∗*^
LDH IU/L	272 (198–435)	304 (232–476)	217 (164–328)	0.000^*∗∗*^	305 (230–487)	242 (180–375)	0.000^*∗∗*^
Anion gap, mmol/L	17.4 (14.5–21.0)	17.0 (14.2–21.3)	18.2 (15.6–20.6)	0.129	17.9 (14.5–22.1)	17.3 (14.4–20.2)	0.012^*∗*^
Lactate^g^, mmol/L	1.9 (1.3–3.1)	1.8 (1.3–2.5)	1.9 (1.4–3.6)	0.033^*∗*^	1.9 (1.4–3.3)	1.8 (1.3–3.1)	0.239
PaO_2_/FiO_2_,_ _mmHg	189.0 (121.8–263.3)	170.5 (109.4–229.9)	208.5 (146.6–294.0)	0.000^*∗∗*^	169.2 (100.6–238.6)	204 (150.0–277.3)	0.000^*∗∗*^
PaO_2_,_ _mmHg	85 (70–119)	80 (68–109)	97 (77–135)	0.000^*∗∗*^	81 (67–115)	87 (73–131)	0.055

Quantitative data was presented as median (IQR), and qualitative data was presented as *n* (%) except otherwise indicated. SD, standard deviation; IPPV, invasive ventilation; NPPV, noninvasive ventilation; ARF, acute renal failure; CRRT, continuous renal replacement therapy; MV, mechanical ventilation; ICU LOS, length of ICU stay; hospital LOS, length of hospital stay; PLT, platelet; LDH, lactate dehydrogenase.

^a^Comparison between pulmonary-sepsis and abdominal-sepsis.

^b^Comparison between one-year survivors and nonsurvivors.

^c^Pulmonary infection was defined as pulmonary infection identified during the whole ICU stay period.

^d^When analyzing the ratio of extubation in the first 24 h of ICU administration, patients without mechanical ventilation were excluded.

^e, f^Patients who died in ICU or lost to follow-up were excluded. There was a total of 338 sepsis patients, 183 of them had pulmonary-sepsis, and 135 had abdominal-sepsis when analyzing 28-day or one-year mortality.

^g^There was a total of 290 sepsis patients with measurement of lactate within the first 24 h; of those 155 had pulmonary-sepsis and 114 had abdominal-sepsis.

^*∗*^ *p* < 0.05.  ^*∗∗*^ *p* < 0.01.

**Table 2:** One-year HR-QoL (EQ-5D) of sepsis survivors and comparison between pulmonary-sepsis and abdominal-sepsis.

Variable	Sepsis responders	Pulmonary-sepsis	Abdominal-sepsis	*p*
	*n* = 209	*n* = 97	*n* = 98	
*Mobility*				
No problems	170 (81.3)	70 (72.2)	91 (92.9)	0.000^*∗∗*^
Some problems	25 (12.0)	18 (18.6)	5 (5.1)	0.004^*∗∗*^
Extreme problems	14 (6.7)	9 (9.3)	2 (2.0)	0.033^*∗*^
*Self-care*				
No problems	183 (87.6)	78 (80.4)	95 (96.9)	0.000^*∗∗*^
Some problems	10 (4.8)	8 (8.2)	1 (1.0)	0.018^*∗*^
Extreme problems	16 (7.7)	11 (11.3)	2 (2.0)	0.010^*∗*^
*Usual activity*				
No problems	169 (80.9)	67 (69.1)	93 (94.9)	0.000^*∗∗*^
Some problems	25 (12.0)	20 (20.6)	3 (3.1)	0.000^*∗∗*^
Extreme problems	15 (7.2)	10 (10.3)	2 (2.0)	0.018^*∗*^
*Pain/discomfort*				
No problems	139 (66.5)	56 (57.7)	72 (73.5)	0.024^*∗*^
Some problems	64 (30.6)	39 (40.2)	23 (23.5)	0.014^*∗*^
Extreme problems	6 (2.9)	2 (2.1)	3 (3.1)	0.505
*Anxiety/depression*				
No problems	169 (80.9)	72 (74.2)	86 (87.8)	0.018^*∗*^
Some problems	35 (16.7)	23 (23.7)	10 (10.2)	0.013^*∗*^
Extreme problems	5 (2.4)	2 (2.1)	2 (2.0)	1.000
*EQ5D index (IQR)*	0.848 (0.729–0.848)	0.768 (0.668–0.848)	0.848 (0.768–0.848)	0.000^*∗∗*^
*EQ-VAS (IQR)*	80 (68.7–90)	75 (60–85)	80 (70–90)	0.001^*∗∗*^

Data was presented as *n* (%). Patients who refused to finish the questionnaire were excluded. ^*∗*^ *p* < 0.05.  ^*∗∗*^ *p* < 0.01.

**Table 3:** Univariate and multivariate regression analysis for risk factors of one-year mortality and quality of life.

Predictors	OR (95% CI)	*p* ^a^	OR (95% CI)	*p* ^b^
A: one-year mortality				
Age	1.036 (1.017–1.043)	0.000^*∗∗*^	1.025 (1.011–1.039)	0.001^*∗∗*^
APACHE II	1.097 (1.067–1.129)	0.000^*∗∗*^	—	—
SOFA	1.159 (1.102–1.219)	0.000^*∗∗*^	—	—
Charlson Comorbidity index	1.068 (1.012–1.126)	0.016^*∗*^	—	—
Malignancy	1.770 (1.034–3.030)	0.045^*∗*^	—	—
Septic shock	2.327 (1.549–3.495)	0.000^*∗∗*^	2.533 (1.525–5.568)	0.000^*∗∗*^
Fungus infection	2.213 (1.424–3.167)	0.000^*∗∗*^	1.846 (1.160–2.938)	0.010^*∗*^
Acute renal failure	3.854 (2.152–6.904)	0.000^*∗∗*^	2.914 (1.525–5.568)	0.001^*∗∗*^
Cystatin c	1.453 (1.193–1.768)	0.000^*∗∗*^	—	—
Anion gap	1.071 (1.031–1.113)	0.000^*∗∗*^	1.070 (1.025–1.117)	0.002^*∗∗*^
Pulmonary infection^c^	2.243 (1.465–3.436)	0.000^*∗∗*^	2.547 (1.513–4.288)	0.000^*∗∗*^

B: one-year QoL				
APACHE II	1.048 (1.008–1.088)	0.017^*∗*^	—	—
Chronic heart failure	6.217 (1.343–28.786)	0.019^*∗*^	—	—
Pulmonary infection^c^	2.939 (1.621–5.329)	0.000^*∗∗*^	2.846 (1.530–5.294)	0.004^*∗∗*^
Tracheal extubation in 24 h^d^	0.231 (0.083–0.645)	0.005^*∗∗*^	0.330 (0.110–0.989)	0.048^*∗*^
Mechanical ventilation days	1.036 (1.009–1.064)	0.008^*∗∗*^	—	—

A: *n* = 435. Variables eliminated from backward selection.

B: *n* = 209. Variables eliminated from backward selection.

^a^Results of univariate analysis.

^b^Results of multivariate analysis.

^c^Pulmonary infection was defined as pulmonary infection identified during the whole ICU stay period.

^d^ *n* = 188; patients without mechanical ventilation were excluded.

^*∗*^ *p* < 0.05.  ^*∗∗*^ *p* < 0.01.

**Table 4:** Baseline characteristics of sepsis survivors with good/poor one-year QoL.

Variables	Good QoL	Poor QoL	*p*
	*n* = 106	*n* = 103	
Age, mean (SD)	54.6 (16.7)	58.8 (16.4)	0.066
Male sex, *n*%	80 (75.5)	63 (61.2)	0.037^*∗*^
APACHE II, mean (SD)	18.1 (7.0)	20.6 (7.6)	0.016^*∗*^
SOFA, mean (SD)	7.2 (3.7)	7.9 (4.3)	0.245
Charlson Comorbidity Index	2 (0,3)	3 (1,4)	0.021^*∗*^
Septic shock, *n*%	60 (56.6)	57 (55.3)	0.890
Chronic heart failure, *n*%	2 (1.9)	11 (10.7)	0.010^*∗*^
Pulmonary infection^a^, *n*%	50 (47.2)	79 (76.7)	0.000^*∗∗*^
Pulmonary-sepsis, *n*%	38 (25.8)	59 (57.3)	0.002^*∗∗*^
ICU treatment within 24 h, *n*%			
IPPV	88 (83.0)	87 (84.5)	0.852
NPPV	3 (2.8)	11 (10.7)	0.028^*∗*^
Tube extubation^b^	18 (20.0)	5 (5.1)	0.003^*∗∗*^
Vasopressor	28 (26.4)	34 (33.0)	0.364
MV, d	6 (22–12)	8 (4–17)	0.003^*∗∗*^
ICU LOS, d	11 (6–19)	15 (7–27)	0.016^*∗*^
Hospital LOS, d	26.5 (16.8–42.3)	31 (20–58)	0.034^*∗*^

Quantitative data was presented as median (IQR), and qualitative data was presented as *n* (%) except otherwise indicated. SD, standard deviation; IPPV, invasive ventilation; NPPV, noninvasive ventilation; MV, mechanical ventilation; ICU LOS, length of ICU stay; hospital LOS, length of hospital stay;

^a^Pulmonary infection was defined as pulmonary infection identified during the whole ICU stay period.

^b^ *N* = 188; patients without mechanical ventilation were excluded.

^*∗*^ *p* < 0.05.  ^*∗∗*^ *p* < 0.01.
