# Supplementary material for: Pulmonary Infection Is an Independent Risk Factor for Long-Term Mortality and Quality of Life for Sepsis Patients
Source: Biomed Res Int. 2016 Dec 5;2016:4213712. doi: 10.1155/2016/4213712 (PMC5165149; doi:10.1155/2016/4213712)
Supplement: Supplementary file 1 — Of the age-matched sepsis cohort, there was no difference in age, Charlson Comorbidity Index and SOFA score. However, pulmonary sepsis had apparently higher APACHE II(22.1 vs 18.6), prolonged mechanical ventilation days, longer ICU and hospital days. Abdominal-sepsis patients were more prone to develop septic shock. [file 4213712.f1.docx]

**Supplementary Table 1. Baseline Characters of the 1:1 age-matched sepsis cohort of ICU survivors**

| Variables | Pulmonary-sepsis  n = 126 | Abdominal-sepsis  n = 126 | *p* |
| --- | --- | --- | --- |
| Age, mean(SD) | 54. 3 (16.2) | 58.5 (16.1) | .693 |
| Male sex, n% | 88 (69.8) | 80 (63.5) | .350 |
| APACHE II, mean(SD) | 22.1 (6.6) | 18.6 (7.6) | .000^**^ |
| SOFA, mean(SD) | 8.4 (3.7) | 7.6 (4.0) | .111 |
| Charlson index | 3 (1,4) | 3 (1,4) | .101 |
| Septic shock, n% | 69 (54.8) | 88 (69.8) | .019^*^ |
| Acute renal failure, n% | 19 (15.1) | 14 (11.1) | .456 |
| Chronic heart failure, n% | 15 (11.9) | 6 (4.8) | .066 |
| MV, d | 11 (5-20) | 5 (2-12) | .000^**^ |
| ICU LOS, d | 15 (8-27) | 9 (4-20) | .001^**^ |
| Hospital LOS, d | 26 (15-39) | 25 (15-44) | .034^*^ |

Quantitative data was presented as median (IQR), and qualitative data was presented as n (%) except otherwise indicated. SD, standard deviation; MV, mechanical ventilation; ICU LOS, length of ICU stay; hospital LOS, length of hospital stay.

^*^ *p* < 0.05.^**^ *p* < 0.01.
